# Introduction of p16^INK4a^ as a surrogate biomarker for HPV in women with invasive cervical cancer in Sudan

**DOI:** 10.1186/s13027-017-0159-0

**Published:** 2017-09-30

**Authors:** Hina Sarwath, Devendra Bansal, Nazik Elmalaika Husain, Mahmoud Mohamed, Ali A. Sultan, Shahinaz Bedri

**Affiliations:** 1 Department of Pathology and Laboratory Medicine, Weill Cornell Medicine – Qatar, Cornell University, Qatar Foundation - Education City, Doha, Qatar; 2 Department of Microbiology and Immunology, Weill Cornell Medicine – Qatar, Cornell University, Qatar Foundation - Education City, Doha, Qatar; 3 Faculty of Medicine, Omdurman Islamic University, P.O. Box 382, Omdurman, Sudan

**Keywords:** Cervical cancer, Human Papillomavirus, p16^INK4a^, Ki-67, Immunohistochemistry, Sudan

## Abstract

**Background:**

Cervical cancer is the fourth most common cancer in women worldwide with highest incidence reported in Eastern Africa in 2012. The primary goal of this study was to study the expression of p16^INK4a^ in squamous cell carcinoma (SCC) of the cervix by immunohistochemistry (IHC) and determine relation with clinico-pathological parameters. This study further explored the correlation of p16^INK4a^ immunostaining with another proliferation marker, Ki-67 and to study if human papillomavirus (HPV) IHC can be used as a marker for detection of virus in high-grade dysplasia.

**Methods:**

A total of 90 samples, diagnosed for cervical cancer, were included in the study. Fixed Paraffin Embedded (FFPE) tissue sections were stained with anti-p16^INK4a^, anti-Ki-67 and anti-HPV antibodies using automated immunohistochemistry platform (ASLink 48-DAKO).

**Results:**

Immunohistochemical protein expression of p16^INK4a^ positivity was found to be highest in SCC (92.2%, *n* = 71) than other HPV tumors (76.9%, *n* = 10). The majority of cases (97.4%) were p16^INK4a^ positive in the age group 41–60 years. In addition, a statistically significant difference between p16^INK4a^ and HPV was observed among total cervical tumor cases and SCC cases.

**Conclusions:**

As expected staining of invasive cervical cancer with anti-HPV showed rare positivity because HPV heralds active infection in dysplastic lesions and not of frank cervical carcinoma. In contrast, anti-p16^INK4a^ IHC results showed positive correlation in SCC and other cervical tumors.

**Electronic supplementary material:**

The online version of this article (doi:10.1186/s13027-017-0159-0) contains supplementary material, which is available to authorized users.

## Background

Cervical cancer continues to be a major public health problem in less developed regions of the world and ranked at fourth position among various cancers in women with estimated age standardized rates over 30 per 100, 000 women [1]. Global prevalence of cervical cancer is estimated 11.7%, out of which Sub-Saharan Africa was reported most affected region with highest prevalence rates (24%) [2]. It has been well documented that developed nations have significantly reduced the incidence and mortality rates of cervical cancer by successful implementation and sustenance of population wide primary screening programs using the cytological Pap smear and HPV DNA testing [3]. In contrast, data published by the WHO / ICO HPV Information Center reports that frequency of cervical cancer rises steadily in Sudan where 2.7% of women from the total population harbor 82–94% of high-risk HPV subtypes 16, 18, 45, 52 and 58 in the age group ranging between 15 and 44 years of age [4–7]. In Sudan, the current and commonest diagnostic method used to detect high-grade cervical dysplasia is the visual inspection with acetic acid (VIA) test [8, 9]. Though, VIA method showed higher sensitivity to detect high-grade lesions than Pap test but it has a lower specificity and misdiagnosis of cervical cancer ranges ~20–50% of true disease [10, 11]. Currently, molecular assay such as HPV DNA testing has been standardized and being commonly used with cytology.

HPV mediated cervical carcinogenesis is a process divided into two stages: productive and transforming infections. Productive infections are short-term lesions, which regress spontaneously, however transforming infections with certain high risk-HPV (HR-HPV) progress to invasive cancer gradually (20–30 years) if not followed up. Histomorphological types of cervical cancers include squamous cell carcinoma (SCC), adenocarcinomas and neuroendocrine carcinomas. SCC represents the majority of cases diagnosed (80%) while the remainder cases are adenocarcinomas and other types [12, 13].

A previously published study from Sudan has reported that women are diagnosed in late stages with aggressive disease emphasizing lack of efficient screening tests [8]. To facilitate early detection and proper treatment, there is a need to evaluate and introduce robust molecular markers of functional biological relevance, which can track disease progression and help in patient stratification [12, 14].

Among the various biomarkers established to facilitate early screening is the cell cycle regulatory protein known as p16^INK4a^, which has demonstrated to be highly sensitive and specific marker of high-grade squamous and glandular neoplasia of the cervix due to its overexpression in cancerous and precancerous cervical lesions [3, 15]. Moreover, p16^INK4a^ inhibits the phosphorylation of pRb mediated through cyclin dependent kinases and regulating mitotic transition of cell cycle from G1 to S phase [16, 17]. Alterations in this host cell proliferation pathway is due to interference of viral oncoproteins E6 and E7 causing erratic cell division which leads to enhanced expression and cellular accumulation of p16^INK4a^ in the basal and parabasal epithelial cells of HPV transformed lesions [18]. A recent meta-analysis study has shown that p16^INK4a^ over-expression using immunohistochemistry (IHC) is linked with increased and disease free survival hence proving to be a marker for prognosis [19]. Studies have also shown that HR-HPV DNA presence is elevated in low-grade cervical lesions indicating active viral multiplication in host cell. Immunostaining of anti-HPV protein expression is detectable in initial transient cervical lesions but not in advanced lesions such as invasive SCC where p16^INK4a^ is over-expressed [20].

In addition, cellular proliferation marker that could be used in conjunction with p16^INK4a^ is Ki-67. HPV infected cells/lesions that overexpressed p16^INK4a^, as a response to viral oncogenes will concomitantly express Ki-67 [18]. Several studies have shown the relation of Ki-67 antibody expression in malignant lesions such as vulva, penis, breast and uterine cervix [21, 22]. Localized in the nucleus, this non-histone cell proliferation antigen is expressed in all cell cycle phases except G0 [23]. Increased Ki-67 expression in the basal layers of the dysplastic epithelium strongly correlates with lesion aggressiveness, and therefore can be used as predicator of proliferation and disease progression [24]. In addition, multiplexing p16^INK4a^ and Ki-67 in both SCC and adenocarcinoma (AC) can be a synergistic approach to quantitate each marker at a cellular level in tumor regions instead of the benign proliferative zones, thereby increasing diagnostic accuracy in both immunohistochemistry studies [25].

The present study aimed to determine the expression of p16^INK4a^ protein in SCC of the cervix by IHC and relate the findings with clinico-pathological characteristics, correlate p16^INK4a^ overexpression with proliferation marker Ki-67 and validate that anti-HPV immunostaining is not suitable for detecting presence of virus in high grade invasive carcinoma.

## Methods

### Study population and samples

The Joint Institutional Review Board (JIRB) of Weill Cornell Medicine - Qatar (WCM-Q) and Hamad Medical Corporation (HMC) Research Office, Qatar has approved the present study (Protocol no.-10,165/10). This is a retrospective cohort study with a total of 90 formalin-fixed paraffin-embedded (FFPE) cervical carcinoma tumors obtained from patients diagnosed at the pathology laboratory at the National Health Laboratory (NHL), Sudan between years 2004–2008. Clinicopathologic information such as age, tumor differentiation and diagnosis was obtained when available.

### Histopathological analysis

All cervical biopsies were fixed in 10% neutral buffered formalin, dehydrated and paraffin embedded. FFPE specimens were sectioned on microtome (4 μm thickness) for both Hematoxylin & Eosin (H&E) staining and immunostaining**.** H&E stained slides were examined for histopathologic diagnosis by experienced pathologist to record morphological alterations such as presence of koilocytotic cells, extent of keratinization and significant differentiation in the squamous epithelial cells (dysplasia).

### Immunohistochemical staining and scoring

Immunostaining of FFPE tissue sections was performed using the Envision Flex™ protocol on ASLink48 (Dako, Glostrup, Denmark), as described previously [26]. The antibody titers and staining parameters were optimized on recommended control tissue according to the manufacturer’s instructions (Dako, Glostrup, Denmark). The antibodies used in this study were: anti-p16^INK4a^ (dilution 1:3, clone E6H4, Roche, Tucson, AZ, USA), anti-HPV (dilution 1:50, clone K1H8, Dako, Glostrup, Denmark) and anti-human Ki-67 (dilution 1:200, clone MIB-1, Dako, Glostrup, Denmark). Briefly, after antigen retrieval the fixed tissue sections were blocked and incubated with primary antibodies for 20 min, washed, and secondary antibody coupled to HRP was added. The reaction was developed using DAB Chromogen (Dako, Glostrup, Denmark) and counterstained with Hematoxylin stain following dehydration in ethanol. Positive and negative tissue controls such as tonsil for p16^INK4a^, Ki-67 and human condyloma tissue for HPV immunostaining were included in each run to validate the stain localization whereas the negative control was the same tissue section omitting primary antibody.

Scoring of immunostained tumor sections was performed by an experienced pathologist. Strong diffuse nuclear as well as cytoplasmic staining in the basal layers of the squamous epithelium is considered p16^INK4a^ positive. Depending on the distribution of the stain in the epithelium, positivity is scored as strong diffuse or high intensity (3^+^), strong focal or moderate intensity (2^+^) and weak sporadic or mild (1^+^). For anti-HPV, positive staining in the nuclei of infected squamous cells and occasional staining of cytoplasm in koilocytotic & dysplastic epithelial cells was observed. All the cases were scored as positive or negative based if protein expression of HPV capsid protein (VP1) was observed. In case of anti-Ki-67 antigen, depending on the intensity and proportion of mitotic cells in the basal and parabasal cells of the squamous epithelium, scores were assigned as follows: <10- Negative, 10–30 cells- 1^+^, 40–60 cells- 2^+^, 70–100- 3^+^.

### Statistical analysis

Immunohistochemical intensity for each of the three markers irrespective of the score was designated as positive for presence and negative if absent. Results were presented using frequencies and percentages. Comparison between different groups was performed using ANOVA test, followed by Student’s t-test. Sensitivity and specificity between p16^INK4a^ and HPV immunostaining were compared using McNemar’s test and positive predictive value (PPV) and negative predictive value (NPV) were recorded. Two tailed test were used for analysis and *p*-value <0.05 was considered significant. The SPSS statistical package (IBM SPSS version 22.0.) was used for the statistical analysis.

## Results

### Demographic and clinical characteristics of the study population

**Fig. 1:**
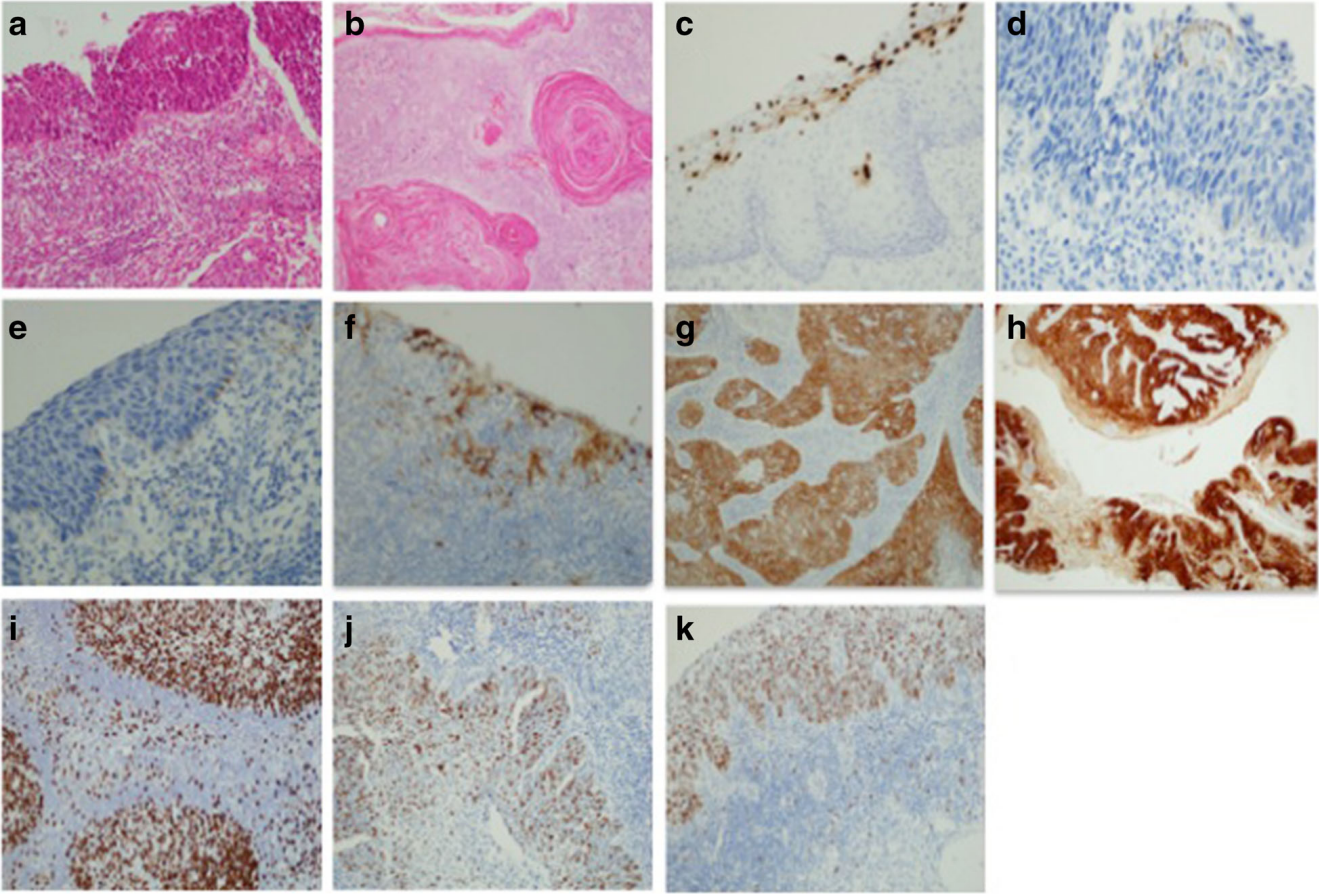
Representative pictures of Hematoxylin & Eosin (H&E) and Immunohistochemical staining for p16^INK4a^, HPV and Ki-67 in squamous cell carcinoma (SCC). **a** Non-keratinizing SCC (H&E) (**b**) Keratinizing SCC (H&E) (**c**) anti-HPV positive control (human condyloma) (**d**) anti-HPV positivity in other tumors (**e**) anti-HPV positivity in SCC (**f**) p16^INK4a^ positive control (tonsil) (**g**) p16^INK4a^ moderate intensity (focal) immunostaining in SCC (**h**) p16^INK4a^ high intensity (diffuse) immunostaining in SCC (**i**) Ki-67 positive control (tonsil) (**j**) Ki-67 proliferating epithelial cells in SCC (**k**) Ki-67 positivity in SCC (**a**-**k**: ×20)

This study includes 90 cases of cervical tumor biopsies from Sudan. Based on histology analysis, data in Table 1, 85.6% (*n* = 77) of women were diagnosed with squamous cell carcinoma (SCC) and 14.4% (*n* = 13) of other HPV associated tumors. In addition, tumor grade, which is the histopathologic differentiation of cancerous tissue, were classified into well differentiated, moderately and poorly differentiated. The age range among the studied population was 27–80 years with a mean age of 54.2 (±13.5) years. Furthermore, the individuals were categorized into 3 age groups (<40, 41–60, >60 years), in which majority (52.1%) of women were aged 41–60 years. However, age was not reported for seven patients in the study (Table 1). The degree of differentiation, an important classification in SCC cases revealed that the majority of samples were of non-keratinizing 68.8% (*n* = 53) subtypes, 26% (*n* = 20) were keratinizing carcinomas. Data were not available for four SCC cases (Table 1 and Fig. 1a, b).

**Table 1:** Demographic, clinico-pathological characteristics and frequency of different immunostaining profiles according to diagnosis among women from Sudan

	SCC *n* = 77^a^ (%)	Other tumors *n* = 13 (%)	*p* value
Age (years)			
21–40	13 (18.6%)	3 (23.1%)	0.134
41–60	36 (51.4%)	3 (23.1%)	
61–80	21 (30.0%)	7 (53.8%)	
Pathological diagnosis			
Keratinizing	20 (26.0)		NA
Non-keratinizing	53 (68.8)		
No data	4 (5.2)	13 (100)	
p16^INK4a^ immunostaining			
Positive	71 (92.2)	10 (76.9)	0.119
Negative	6 (7.8)	3 (23.1)	
HPV immunostaining			
Positive	7 (9.1)	2 (15.4)	0.384
Negative	70 (90.9)	11 (84.6)	
Ki-67 immunostaining			
Positive	47 (61.0)	8 (61.5)	0.314
Negative	5 (6.5)	2 (15.4)	
No data	25 (32.5)	3 (23.1)	

^a^Age of 7 patients were not available

### Immunostaining

**Fig. 2:**
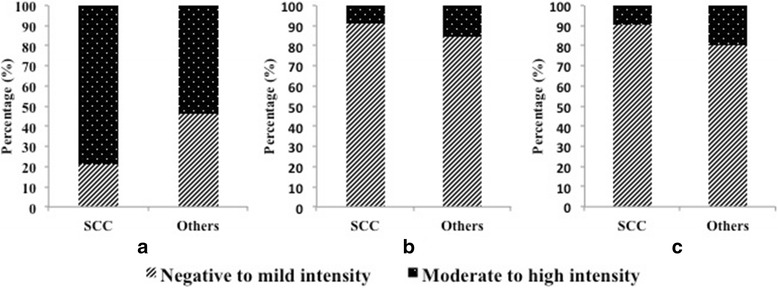
Differential staining of (**a**) p16^INK4a^, (**b**) HPV and (**c**) Ki-67 between SCC and other tumors

Immunohistochemistry (IHC) of FFPE tissue samples was performed using anti-p16^INK4a^, Ki-67 and anti-HPV antibodies (Fig. 1c-k). Overexpression of p16^INK4a^ was found to be higher in SCC, 92.2% (n = 71) compared to other HPV tumors, 76.9% (n = 10) (Table 1). Furthermore, based on immunostaining intensity, we extended our analysis to evaluate and compare the test sensitivity between different immunostaining and diagnosis, we found a significant difference in p16^INK4a^ among SCC cases (sensitivity = 79.2%, specificity = 46.1%, PPV = 83.9%, NPV = 27.2%, *p* = 0.049) (Fig. 2a). However, no significant difference was observed in HPV and Ki-67 immunostaining (*p* = 0.484 and *p* = 0.329), respectively (Fig. 2b, c). Additionally, an exact McNemar’s test determined that there was a statistically significant difference between p16^INK4a^ and HPV among cervical tumor cases (*p* = 0.011) and SCC cases alone (p = 0.049) however; no significant difference was noted in other tumor cases (*p* = 0.219) (Table 2). Furthermore, no significant correlation was found between tumor grade and p16^INK4a^ immunostaining.

**Table 2:** Distribution of p16^INK4a^ versus HPV immunoexpression in 90 cervical tumors cases according to diagnosis in women from Sudan

Diagnosis	HPV	p16^INK4a^		*p* value^£^
		Negative n (%)	Positive n (%)	
SCC	Positive	3 (18.8)	4 (6.6)	0.049﻿^£﻿^
	Negative	13 (81.2)	57 (93.4)	
Others	Positive	1 (16.7)	1 (14.3)	0.219
	Negative	5 (83.3)	6 (85.7)	
Total	Positive	4 (18.2)	5 (7.4)	0.011^£^
	Negative	18 (81.8)	63 (92.6)	

^£^significant

**Fig. 3:**
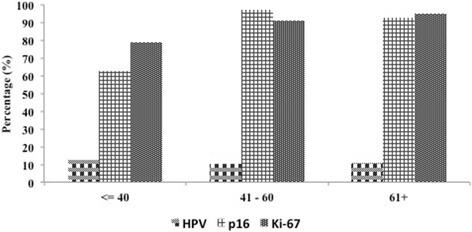
Percentage of HPV, p16 ^INK4a^ and Ki-67 immunostaining according to age group. (*p = 0.001)

Of the total 90 samples collected in this study, Ki-67 immunoreaction was performed only in 68.9% (*n* = 62) cases (Table 1). Of these, 88.7% (*n* = 55) were found to be positive for Ki-67 immunostaining (Table 1). Furthermore, to correlate expression of p16^INK4a^ with a proliferation marker, Ki-67 according to the diagnosis, a significant difference was observed in other HPV associated tumors (*p* = 0.019) however, no significant association was found with SCC cases (Additional file 1: Table S1). According to age groups, the percentage frequency of p16^INK4a^ was significantly higher in the age group 41–60 years (*p* = 0.001), however, no association with age group was seen in Ki-67 and HPV immunostaining (Fig. 3).

## Discussion

Meta-analysis studies have reported that cervical cancer incidence and mortality rates are rising in sub- Saharan Africa with an estimated prevalence of oncogenic HPV genotypes across all ages at 33.6% specifically in Eastern Africa [2]. The HPV distribution data in Africa reveals two peaks, one at <25 and another ≥45 years of age [2]. In the present study, we observed that 52.1% of women in age group between 41 and 60 years were diagnosed with SCC, a dominant histomorphological form in invasive cervical cancer [12]. According to the WHO guidelines, all women between ages of 35–45 years must be screened annually, which can significantly reduce mortality rates [27]. The degree of differentiation (keratinization) is an important pathological feature in cervical tumors, indicates the site of infection in the cervical mucosal epithelium (squamo-columnar junction) and susceptible to HPV infection. In this study, majority of SCC cases were classified as non-keratinizing SCC, 68.8% and 26% were keratinizing SCC, this corroborates previous findings from Ethiopia [28].

Correlating p16^INK4a^ expression with clinico-pathological parameters, we found that p16^INK4a^ expression was directly proportional to the diagnosis of cervical tumors where 90.1% of SCC cases and 76.9% as Adenocarcinoma (AC) showed positive p16^INK4a^ expression, which is in agreement with previous studies [15, 29, 30]. However, Lin J et al. [19], reports that prognostic significance of p16^INK4a^ overexpression was not significantly associated with tumor grade nor tumor size, however p16^INK4a^ overexpression was highly associated with better prognosis and increased disease-free survival which, corroborates with previous studies [31, 32]. Additionally, in the present study, 9.9% (*n* = 7) of SCC cases and 23.1% (*n* = 3) of AC cases were p16^INK4a^ negative which has also been reported in previous studies [33, 34, 35]. Furthermore, absence of p16^INK4a^ expression in the cervical tumors does not indicate lack of HR- HPV or improper IHC technique but rather implicated due to silencing of the gene through mutations in the promoter regions resulting in transcription failure, epigenetic mechanisms and hyper methylation.

Molecular assays are the most sensitive for HPV detection in FFPE samples than IHC [32]. In the present study, the frequency of both p16^INK4a^ and HPV positive expression was seen in age group between 41 and 60 years (n = 3, 3.6%). The positive correlation of HPV expression with p16^INK4a^ intensity and dispersion on cervical carcinoma cases was reported in a study in Saudi Arabia [30]. However, majority (91.5%) of SCC cases were positive for p16^INK4a^, but negative for HPV-1 derived capsid protein (VP1). The majority of cervical cancers does not have active dysplasia and therefore are limited in protein expression of HR-HPV.

To increase the reproducibility of diagnosis, p16^INK4a^ expression was compared with a cellular proliferation marker, Ki-67. To date, several studies have shown co-expression of p16^INK4a^ and Ki-67 when combined concurrently improve diagnostic accuracy and that Ki-67 expression increases linearly with tumor grade in SCC [25, 34, 36]. In this study, ~61% of SCC and AC cases were positive for Ki-67. Furthermore, the frequency of p16^INK4a^ and Ki-67 positive immunostaining when assessed individually was higher in the age group >40 and 6.6% (*n* = 4) were both positive for p16^INK4a^ and Ki-67. Several studies have shown that both p16^INK4a^ and Ki-67 proteins are co-expressed in most high-grade squamous lesions, which is also observed in this study. Ki-67 is employed as an objective marker indicating aberrant cellular proliferation that facilitates measurement of advanced disease end point, which is progress of CIN to carcinoma. Hence, direct proportionality in Ki-67 expression in relation to tumor grade along with p16^INK4a^ is involved functionally in the process of HPV induced transformation and overexpressed in the epithelium [29, 36]. Furthermore, Ki-67 expression was found to be significantly different in cervical tumor lesions CIN2 and CIN3 [33] and also expressed in benign proliferative lesions and in basal cells of normal squamous mucosa [25].

In the present study, no correlation was found between p16^INK4a^ and Ki-67 with respect to HPV immunohistochemical protein expression, which is in agreement with previous report [29]. HPV detection rate using in situ hybridization (ISH) varied significantly between cervicitis and low grade SIL; in addition, significant correlation of HR-HPV status with histopathological grade was observed [37]. In contrast, p16INK4a and Ki67 was found useful to detect both LR and HR-HPV in precancerous lesions and distinguish between low grade SIL and high grade SIL [38].

Based on the tumor grade, it has been shown that in low-grade lesions, p16^INK4a^ is diffusely expressed in ~60% of CIN1 and mostly associated with HR-HPV genotypes, but HR-HPV presence was detected in p16^INK4a^ negative tumors [36, 39]. In addition, high incidence of HR-HPV has been observed among young women and most of these infections were transient and regress spontaneously [39]. We therefore concluded that p16^INK4a^ overexpression is already indicative of advanced viral interference progressing towards invasive cancer where aside from active viral replication there is significant morphological change occurring at histology, cellular and molecular level which can be visually scored. However, in invasive cervical cancer, the HPV protein expression was not detectable by IHC and HPV genotyping in FFPE tumor samples.

Currently, in Sudan precancerous cervical lesions are visually detected by VIA [8–10] unlike the Pap test, a primary screening method in industrialized nations, VIA has limitations to distinguish early morphological changes associated with neoplastic transformation. However, Pap test has not been implemented as a screening modality in low resource settings with no national HPV vaccination program [4]. Despite the increasing cancer related incidence and mortality rates, Sudan is burdened with other public health issues such as malaria, leprosy, tuberculosis, HIV/AIDS thereby causing lack of emphasis and knowledge about this disease [40]. Thus, introduction of such molecular biomarkers will prove beneficial in early detection and embarking on screening initiatives and reducing mortality [27]. Recently, WHO has recommended that HPV vaccination should be performed as part of national immunization programs for women between ages 13–26 years to effectively prevent disease [27]. In addition, it was suggested that boys should be included in HPV vaccination programs to overcome cost effectiveness in low resource settings [41]. Current treatment for invasive cervical cancer in sub-Saharan Africa is radiotherapy, which is a major challenge due to lack of both resources and primary screening facilities hence, resulting in progress to advanced disease stage when diagnosed [8, 40]. Thus, there is a need to establish frequent screening intervals of optimal target population as progression from low grade to invasive cervical cancer takes up to 20 years [27] and adopt algorithm models for cervical cancer prevention in developed countries to help in proper management of disease [41].

The strength of the present study lies in the use of IHC technique to study immunochemical staining along with H&E stain of SCC tumors for histopathology assessment. The monoclonal antibody clone used for p16^INK4a^-E6H4 has been recommended specific antibody for exfoliated dysplastic cells both in histology tissue sections and cytology smears and that normal cervical epithelium, inflammatory or metaplastic lesions were not stained [14].

Limitations of this study are primarily the tissue quality, preservation and fixation of FFPE samples were not suitable to carry out analysis for HPV genotyping study. In addition, cut-off threshold for immune-positivity of p16^INK4a^ varies between pathologists, presenting a challenge as no standard parameters. Limited sample number for Ki-67 hence proper correlation with p16^INK4a^ in SCC cases could not be made. Larger cohort with proper diagnostic, survival data can help to correlate other clinical parameters.

## Conclusions

In this preliminary study, we evaluate the clinical utility of p16INK4a as a surrogate marker using IHC technique in invasive cervical carcinoma among women in Sudan. Overall, p16^INK4a^ overexpression in squamous cell carcinoma was significant and differs from expression in other cervical tumors (AC). The immunostaining of p16^INK4a^ and HPV vary in the total sample positive and negative reaction, indicating that p16^INK4a^ is an effective way to detect invasive carcinoma than HPV. Proliferation marker, Ki-67 showed significant correlation with p16^INK4a^ in other HPV associated tumors. Lastly, significant correlation of p16^INK4a^ percentage frequency in age group 41–60 years where active advance transforming CIN occurs concludes that it is an appropriate surrogate marker to help in early screening of cervical neoplasia.

## Additional file

Additional file 1: Table S1. Frequency of p16^INK4a^/Ki-67 immunostaining according to diagnosis and HPV infection in the 63 cervical tumor cases. (DOCX 51 kb)

## Abbreviations

AC: Adenocarcinoma
CIN: Cervical Intraepithelial Neoplasia
FFPE: Formalin-fixed paraffin embedded
H&E: Hematoxylin & Eosin
HMC: Hamad Medical Corporation
HPV: Human Papillomavirus
HR: High risk
IHC: Immunohistochemistry
LR: Low risk
NPV: Negative predictive value
PPV: Positive predictive value
SCC: Squamous cell carcinoma
SIL: Squamous Intraepithelial lesion
SPSS: Statistical package for the Social science
VIA: Visual inspection with acetic acid
WCM-Q: Weill Cornell Medicine-Qatar
