# Supplementary material for: Introduction of p16INK4a as a surrogate biomarker for HPV in women with invasive cervical cancer in Sudan
Source: Infect Agent Cancer. 2017 Sep 30;12:50. doi: 10.1186/s13027-017-0159-0 (PMC5622439; doi:10.1186/s13027-017-0159-0)
Supplement: Additional file 1: Table S1. — Frequency of p16INK4a/Ki-67 immunostaining according to diagnosis and HPV infection in the 63 cervical tumor cases. (DOCX 51 kb) [file 13027_2017_159_MOESM1_ESM.docx]

**Supplementary Table 1: Frequency of p16 ^INK4a^/Ki-67 immunostaining according to diagnosis and HPV infection in the 63 cervical tumor cases**

| **Diagnosis** | **HPV** | **p16/Ki-67** | | | | **p value*** |
| --- | --- | --- | --- | --- | --- | --- |
|  |  | **+ve/+ve** | **-ve/-ve** | **+ve/-ve** | **-ve/+ve** |  |
| SCC | Positive | 4 (100) | 0 | 0 | 0 | 0.674 |
|  | Negative | 40 (87.0) | 0 | 5 (100) | 3 (60.0) |  |
| Others | Positive | 0 | 0 | 1 (100) | 0 | 0.019* |
|  | Negative | 6 (13.0) | 1 (100) | 0 | 2 (40.0) |  |
| Total | Positive | 4 (8.0) | 0 | 1 (16.7) | 0 | 0.771 |
|  | Negative | 46 (92.0) | 1 (100) | 5 (83.3) | 5 (91.9) |  |

*significant (Chi-square test)
